# Greenhouse Gas Emissions Associated With the Mexican Diet: Identifying Social Groups With the Largest Carbon Footprint

**DOI:** 10.3389/fnut.2022.791767

**Published:** 2022-03-31

**Authors:** Nancy López-Olmedo, Dalia Stern, Maryia Bakhtsiyarava, Carolina Pérez-Ferrer, Brent Langellier

**Affiliations:** ^1^Center for Population Health Research, National Institute of Public Health, Cuernavaca, Mexico; ^2^National Council for Science and Technology, Mexico City, Mexico; ^3^Institute of Urban and Regional Development, University of California, Berkeley, Berkeley, CA, United States; ^4^Center for Nutrition and Health Research, National Institute of Public Health, Cuernavaca, Mexico; ^5^Department of Health Management and Policy, Dornsife School of Public Health, Drexel University, Philadelphia, PA, United States

**Keywords:** greenhouse gas emissions, diet, social groups, carbon footprint, Mexico

## Abstract

**Background:**

Most studies of the climate footprint of diets have been conducted in countries in the global north, but the majority of the world population lives in global south countries. We estimated total dietary greenhouse gas emissions (GHGE) in Mexico, examined the contribution of major food and beverage groups, and assessed variation across social groups.

**Methods:**

We linked individual-level dietary data from the Mexican National Health and Nutrition Survey 2018 to the SHARP Indicators Database, containing GHGE estimates for 182 primary food and beverages.

**Results:**

Mean dietary GHGE was 3.9 kg of carbon dioxide equivalent per person per day. Dietary GHGE is highest among those in young adulthood and middle age versus adolescents and older adults, and among males, those with higher educational attainment, higher socioeconomic status, that do not speak an indigenous language, and that live in urban areas.

**Conclusion:**

The Mexican diet has a much lower carbon footprint than diets in other Latin American countries for which such estimates are available. In contrast to patterns observed in Argentina and Brazil, dietary GHGE was lowest in those in lower socioeconomic and educational strata and in rural areas. A better understanding of the differences in diet sustainability between and within countries will be needed for developing global and local strategies that meet the environmental sustainability goals.

## Introduction

The global food supply chain contributes 34% of human-generated greenhouse gas emissions (GHGE), or about 18 billion metric tons of carbon dioxide equivalents (1). As part of the Paris Agreement, 186 countries submitted carbon reduction targets intended to reduce global climate change. The large environmental footprint of food suggests that meeting these targets will require changes across the food system by optimizing production techniques and moderating consumption of climate unfriendly foods.

Realignment toward more climate-friendly diets is also necessary because foods vary considerably in their environmental impact (2, 3). On average, producing one kilogram of beef or lamb results in about 4.5 times more carbon dioxide equivalents than the same amount of pork, eight times more than chicken or fish, and 60 times more than field-grown fruits or vegetables (2). Several studies have evaluated the environmental impact of diets by linking data from life cycle analyses (LCA)^1^ to individual-level dietary data or consumption patterns recommended by a particular diet (e.g., the Mediterranean diet). However, the vast majority of studies have been conducted in countries in the global north (4). While global north countries are responsible for ∼92% of excess carbon dioxide emissions (5), evaluating the environmental impact of diets in countries in the global south, including in the Latin American region, is also important. The majority of the world population lives in the global south, and many of these countries are in the midst of a nutrition transition that will likely have an enormous impact on future GHGE.

In this study, we examine the carbon footprint of diets in Mexico, the second most populous country in the Latin American region. We link dietary data from a large, nationally representative survey to LCA data to determine the mean GHGE associated with the Mexican diet. These findings are an important supplement to similar studies in other Latin American countries (6–8) that differ from Mexico in dietary patterns and population socio-demographics. We also examine GHGE of diet by individual-level socio-demographic characteristics. Previous studies reported that diet quality is better in adults with low versus high socioeconomic status (9). Specifically, Mexican adults in higher socioeconomic categories consume higher percentage of consumers of processed meats, fast food, snacks, sweets and desserts, and sweet cereals (10). These findings are useful for assessing the contributions of different social groups to the overall carbon footprint of diet in Mexico and understanding how GHGE may change in the future as Mexico’s population grows and becomes more urbanized.

## Materials and Methods

### Data Sources

We use two main data sources: first, data regarding dietary intake among the Mexican population is from the 7-day semiquantitative food frequency questionnaire from the 2018 National Health and Nutrition Survey (ENSANUT; from its Spanish acronym, Encuesta Nacional de Salud y Nutrición) (11). The second data source is the SHARP Indicators Database (SHARP-ID), which contains GHGE estimates for 182 primary products (e.g., bananas, milk, beef) (12).

National Health and Nutrition Survey data are collected among a large probability sample of Mexican households designed to be representative at the state level and by urbanicity strata (i.e., rural localities with <2,500 inhabitants and urban localities with ≥2,500 inhabitants) (13). Of the 21,479 participants ≥12 years old that completed the food frequency questionnaire, we excluded 390 (1.8%) that reported implausible consumption levels and an additional 791 (3.7%) pregnant women. Therefore, the analytical sample was composed of 20,298 adolescents and adults.

The food frequency questionnaire used in ENSANUT has been previously validated (14) and is included as Supplementary File 1. Generally, participants report frequencies of consumption for 163 total items (e.g., bananas) in the following food groups: (1) dairy, (2) fruit, (3) vegetables, (4) fast food, (5) pork, (6) beef, (7) processed meat, (8) chicken and eggs, (9) fish and seafood, (10) legumes, (11) grains and tubers, (12) corn-based products, (13) drinks, (14) snacks, sweets, and desserts, (15) soups, creams, and pastas, (16) miscellaneous, (17) tortillas. Our choice of food groups largely tracks with groups used by Mexico’s National Institute of Public Health (INSP) in the food frequency questionnaire. The main exception is that we split the meat and eggs group by animal origin due to the large difference in GHGE between beef and other meat products. For ease of interpretation, we also analyzed food groups in the following aggregated groups: (1) animal products: pork, beef, processed meat, chicken and eggs, dairy; (2) plant-based products: fruit, vegetable, legumes, grains, tubers, tortillas; (3) composite: fast food, corn-based products, snacks, sweets and desserts, soups, creams, pastas; (4) drinks; and (5) miscellaneous.

For each food group, participants report the following with respect to the 7 days prior to the interview: number of days the food item is consumed (i.e., 0–7), the typical times per day the item is consumed, the portion size, and the number of portions consumed per eating occasion. For most food items, portion sizes are reported in standard units (e.g., the standard size for milk is 240 ml). We used liquid densities to convert liquid volume to weight and converted weekly to daily intake to better align with other studies (6, 7, 15, 16).

As described by Mertens et al., SHARP-ID was developed using life cycle inventory data from the Agri-footprint 2.0, Ecoinvent 3.3, and CAPRI databases (12). SHARP-ID was developed to quantify the environmental impact of diets in four European countries – Denmark, Czechia, Italy, and France. GHGE values for each food item are reported in kilograms of carbon dioxide equivalent per kilogram of food as eaten and include impacts from packaging, transport, home preparation (edible portion and cooking process) and food losses and waste.

To determine dietary GHGE, we linked each of the 163 food and beverage items reported in the ENSANUT food frequency questionnaire to the most appropriate item in the SHARP-ID. In most cases, the match was exact. However, in some cases, we matched a more-specific food group from the food frequency questionnaire (e.g., chicken breast) to a slightly broader group in the SHARP-ID (e.g., chicken fresh meat). For composite food items comprised of more than one ingredient (e.g., tamales), we used standard recipes developed by INSP to calculate the GHGE. In total, we matched or calculated GHGE values for 157 of the 163 foods and beverages in the ENSANUT food frequency questionnaire using data from the SHARP-ID. We used values reported in Clune et al. for bananas, fried bananas, and guava and in Guzmán-Soria et al. for three different types of tortillas (2, 17). The linked database with GHGE values for each ENSANUT food and beverage item is included as Supplementary File 2.

### Dietary Greenhouse Gas Emissions

The outcome variable is dietary GHGE, measured in kilograms of carbon dioxide equivalent per capita per day (kg CO_2_-eq/cap/d). We calculated the total dietary GHGE for each participant, as well as the GHGE associated with consumption of each food group. To do this, we first calculated the GHGE attributable to each of the 163 food and beverage products reported in ENSANUT as follows:


$$\mathrm{daily}\,\mathrm{GHGE}=\frac{\left(\frac{\mathrm{days}\,\mathrm{consumed}}{\text{week}}\,\times \,\frac{\mathrm{times}\,\mathrm{consumed}}{\text{day}}\,\times \,\frac{\text{portions}}{\mathrm{time}\,\mathrm{consumed}}\times \frac{\mathrm{kg}\,\mathrm{food}}{\text{portion}}\,\times \,\frac{\mathrm{kg}\,{\mathrm{CO}}_{2}-\text{eq}}{\mathrm{kg}\,\mathrm{food}}\right)}{7\,\mathrm{days}}$$

We then summed the product-level GHGE values across all 163 food and beverage products to determine the total dietary GHGE for each participant. Similarly, we summed the values for every food and beverage product (e.g., milk, cheese) in each food group (e.g., dairy) to determine group-level GHGEs.

### Energy Intake

We include energy intake in calories as a secondary variable. We estimated the average daily energy intake of each food or beverage item using the food composition database compiled by the PI–DIETA network (18). We used a similar approach to the one described above to calculate the total energy intake for each participant (i.e., we summed intake for all reported foods) and energy intake for each food group (i.e., we summed all items in a given group). Though energy intake is reported elsewhere, we present the data here because it is useful for assessing whether any differences in dietary GHGE are due to differences in the quantity of intake.

### Social Characteristics

We considered the following characteristics: age, sex (men/female), educational attainment, socioeconomic status, speaking an indigenous language (yes/no), urbanicity, and region. We classified age into four categories: 12–17 years, 18–29 years, 30–59 years, and 60 years or more. We defined educational attainment as primary school or less, middle school, high school, and college or more. We used a previously developed socioeconomic index constructed using principal components analysis applied to household characteristics and assets. The index was classified into three categories (low, medium, and high) using the tertiles of the distribution of the index as cut-off points (11). The ENSANUT 2018 defines rural areas as locations with <2,500 inhabitants and urban areas as locations with ≥2,500 inhabitants. The country’s regions are defined as North, Central, Mexico City, and South.

### Statistical Analysis

We conducted all analyses using Stata 14. We used sampling weights and design variables to account for differential probability of selection into the sample, differential non-response, and the complex, multi-stage design. We report the means of dietary GHGE and energy intake for the total diet and for each food or beverage group. The total dietary GHGE summarizes the average per capita carbon footprint of the Mexican diet, while total calories summarize energy intake. We also report means of total dietary GHGE and total energy intake stratified by the social characteristics described. We further estimated the ratio of total dietary GHGE to energy intake (per 1,000 Kcal), overall and by sociodemographic characteristics, to capture differences in GHGE net of differences in energy intake. We used energy intake per 1,000 kcal merely as a proxy of the contribution for each food group to total dietary intake and not to assess diet quality. These results are useful for assessing variation in carbon footprints and energy intake across segments of the population. Finally, we report the absolute and relative contribution of each aggregated food and beverage group to total GHGE, stratified by socioeconomic status, sex, and urbanicity. The results with disaggregated food and beverage groups are presented in Supplementary File 3.

### Ethics Approval and Consent to Participate

The 2018 ENSANUT was conducted according to the guidelines laid down in the Declaration of Helsinki and all procedures involving human subjects/patients were approved by the Ethics Committee of the Mexican National Institute of Public Health. Written informed consent was obtained from all subjects under study. All the information used in the present study was obtained from de-identified secondary data.

## Results

Table 1 includes the total dietary GHGE and energy intake, as well as the specific contribution of each food and beverage group. Mean dietary GHGE was 3.9 kg CO_2_-eq/cap/d. The largest contributor to dietary GHGE was beef – which was responsible for 15% of the total dietary GHGE. In contrast, beef contributed only 2% of daily calories. Other large contributors to GHGE were corn products (12%), dairy (11%), beverages (11%), soups, creams, and pastas (9%), and chicken and eggs (9%). Tortillas contributed 23% of calories but only 2% of GHGE, while grains and tubers contributed 10% of calories but just 2% of GHGE.

**TABLE 1:** Dietary greenhouse gas emissions and energy intake per day by food group among the Mexican population.

	Dietary GHGE (kg CO_2_-eq/cap/day)		Energy intake (Kcal/day)	
	Mean (95% CI)	% of total	Mean (95% CI)	% of total
Total	3.9 (3.84, 3.97)	100.0	1907 (1884, 1931)	100.0
Tortillas	0.08 (0.08, 0.09)	2.1	430 (421, 440)	22.5
Drinks	0.41 (0.4, 0.42)	10.5	246 (240, 253)	12.9
Corn products	0.47 (0.46, 0.49)	12.1	194 (188, 199)	10.2
Grains and tubers	0.08 (0.07, 0.08)	2.1	188 (183, 194)	9.9
Dairy	0.44 (0.43, 0.45)	11.3	140 (136, 143)	7.3
Snacks, sweets, and desserts	0.22 (0.2, 0.23)	5.6	125 (121, 129)	6.6
Fast food	0.24 (0.21, 0.27)	6.2	119 (110, 128)	6.2
Chicken and eggs	0.33 (0.32, 0.34)	8.5	111 (109, 114)	5.8
Fruit	0.11 (0.1, 0.11)	2.8	97 (94, 99)	5.1
Vegetables	0.15 (0.14, 0.15)	3.8	59 (57, 62)	3.1
Legumes	0.05 (0.05, 0.05)	1.3	43 (42, 44)	2.3
Beef	0.58 (0.56, 0.61)	14.9	41 (39, 42)	2.1
Pork	0.14 (0.13, 0.15)	3.6	29 (28, 31)	1.5
Miscellaneous	0.05 (0.05, 0.06)	1.3	27 (26, 28)	1.4
Processed meat	0.11 (0.11, 0.11)	2.8	25 (23, 26)	1.3
Soups, creams, and pastas	0.34 (0.32, 0.35)	8.7	23 (22, 23)	1.2
Fish and seafood	0.1 (0.09, 0.11)	2.6	12 (11, 12)	0.6

*GHGE, greenhouse gas emissions. Units are kilograms of carbon dioxide equivalent per capita per day. Rows are sorted by percent contribution to total energy intake.*

Table 2 shows total dietary GHGE, energy intake, and the ratio of total dietary GHGE to energy intake (per 1,000 Kcal) across a range of social groups. Generally, GHGE is highest among those in young adulthood and middle age than adolescents and older adults, as well as among males, those with higher educational attainment, higher socioeconomic status, that do not speak an indigenous language, that live in urban areas, and that live in regions other than the South, particularly in Mexico City and the North. These differences in dietary GHGE persist after adjusting for energy intake, except by sex and age group. Dietary GHGE per 1,000 kcal was higher in men than women and among older versus younger adults. The difference in GHGE between urban versus rural areas is also much greater than the difference in energy intake.

**TABLE 2:** Total dietary greenhouse gas emissions and energy intake per day among the Mexican population, by socio-demographic characteristics.

	Dietary GHGE (kg CO_2_-eq/cap/day)			Energy intake (Kcal/day)			Dietary GHGE per 1000 Kcal		
	Mean	95% CI	*p*-value	Mean	95% CI	*p*-value	Mean	95% CI	*p*-value
Age			<0.0001			<0.0001			<0.0001
12–17	3.60	(3.49, 3.71)		1900	(1853, 1947)		1.93	(1.89, 1.96)	
18–29	4.45	(4.27, 4.64)		2139	(2079, 2200)		2.10	(2.06, 2.14)	
30–59	3.96	(3.88, 4.05)		1926	(1894, 1957)		2.09	(2.06, 2.12)	
≥60	3.36	(3.25, 3.48)		1599	(1561, 1637)		2.13	(2.08, 2.19)	
Sex			<0.0001			<0.0001			<0.0001
Male	4.43	(4.32, 4.54)		2244	(2206, 2282)		1.99	(1.96, 2.02)	
Female	3.44	(3.37, 3.51)		1615	(1593, 1638)		2.15	(2.12, 2.18)	
Educational attainment						<0.0001			<0.0001
Primary school or less	3.16	(3.08, 3.24)		1723	(1687, 1758)		1.88	(1.85, 1.91)	
Middle school	3.78	(3.69, 3.87)		1950	(1912, 1987)		1.98	(1.95, 2.01)	
High school	4.31	(4.19, 4.43)		2015	(1969, 2061)		2.18	(2.13, 2.23)	
College or more	4.94	(4.69, 5.18)		2011	(1934, 2088)		2.48	(2.43, 2.52)	
Socioeconomic status			<0.0001			0.8269			<0.0001
Low	3.28	(3.19, 3.36)		1904	(1869, 1938)		1.75	(1.72, 1.78)	
Medium	3.84	(3.74, 3.94)		1917	(1880, 1954)		2.04	(2.01, 2.07)	
High	4.45	(4.32, 4.58)		1902	(1859, 1945)		2.36	(2.33, 2.4)	
Speaks indigenous language			<0.0001			<0.0001			<0.0001
No	3.96	(3.89, 4.03)		1916	(1892, 1940)		2.10	(2.08, 2.12)	
Yes	2.90	(2.74, 3.06)		1754	(1686, 1822)		1.68	(1.62, 1.75)	
Urbanicity			<0.0001			0.5477			<0.0001
Urban	4.07	(4, 4.15)		1904	(1877, 1932)		2.17	(2.15, 2.2)	
Rural	3.28	(3.18, 3.37)		1919	(1881, 1956)		1.72	(1.68, 1.75)	
Region			<0.0001			0.0015			<0.0001
North	4.17	(4.06, 4.28)		1857	(1819, 1896)		2.27	(2.23, 2.3)	
Central	3.92	(3.83, 4.01)		1958	(1923, 1994)		2.03	(1.99, 2.06)	
Mexico City	4.26	(3.97, 4.54)		1862	(1768, 1955)		2.32	(2.25, 2.39)	
South	3.51	(3.43, 3.59)		1912	(1879, 1945)		1.87	(1.84, 1.9)	

*GHGE, greenhouse gas emissions. Units are kilograms of carbon dioxide equivalent per capita per day. p-values are from F-tests of separate unadjusted linear regression models of the outcome on each social characteristic.*

**FIGURE 1:**
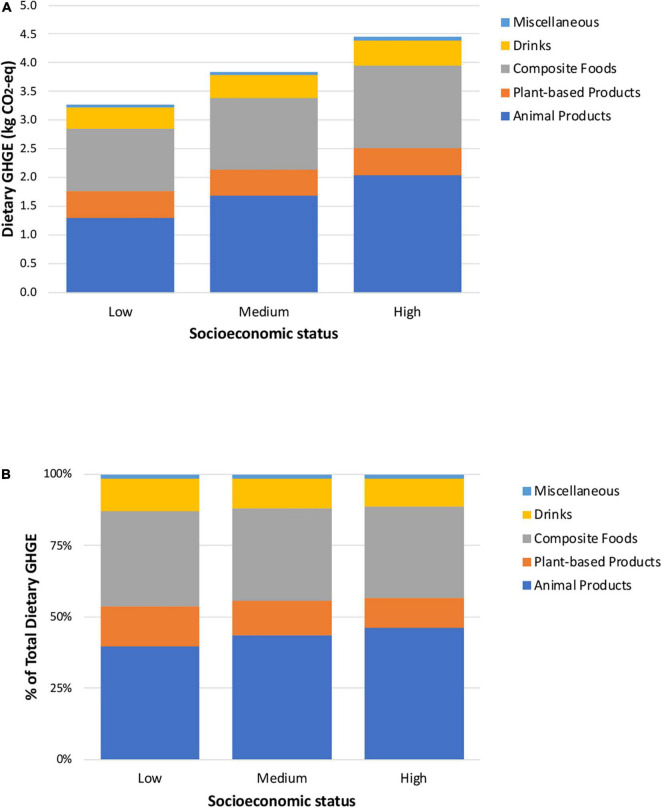
Absolute **(A)** and relative **(B)** contribution of each aggregated food and beverage group to total dietary greenhouse gas emissions, stratified by socioeconomic status.

Figure 1 shows the absolute and relative contribution of emissions associated with each food and beverage group to total dietary GHGE, stratified by level of socioeconomic status. Figure 1A shows that the diets of adolescents and adults in higher versus lower socioeconomic status produce more GHGE for all food and beverage groups. Results of the disaggregated groups show that the greater total dietary GHGE among those with high socioeconomic status are due to higher group-level GHGE for several food groups, including dairy (0.52 high versus 0.34 low), fast food (0.35 high versus 0.10 low), snacks, sweets, and desserts (0.29 high versus 0.14 low), and particularly beef (0.78 high versus 0.37 low) (Supplementary Figure 1A). In relative terms, animal products make a larger contribution to dietary GHGE among those with high (46%) rather than medium (44%) and low (40%) socioeconomic status (Figure 1B). Among the animal food products, beef is the main contributor to GHGE, being higher in adolescents and adults with high socioeconomic status than in those with medium and low socioeconomic status (Supplementary Figure 1). The second largest contributor to GHGE is composite foods, in both absolute and relative terms. Even though its absolute contribution is higher among higher socioeconomic groups (Figure 1A), its relative contribution is similar across socioeconomic strata (Figure 1B). However, the analysis of the disaggregated groups shows that fast food is responsible for a higher proportion of GHGE among the high (8%) than the low (3%) stratum. In contrast, corn-based products contribute more to the low (16%) stratum than the medium (12%) and high (10%) strata (Supplementary Figure 1).

**FIGURE 2:**
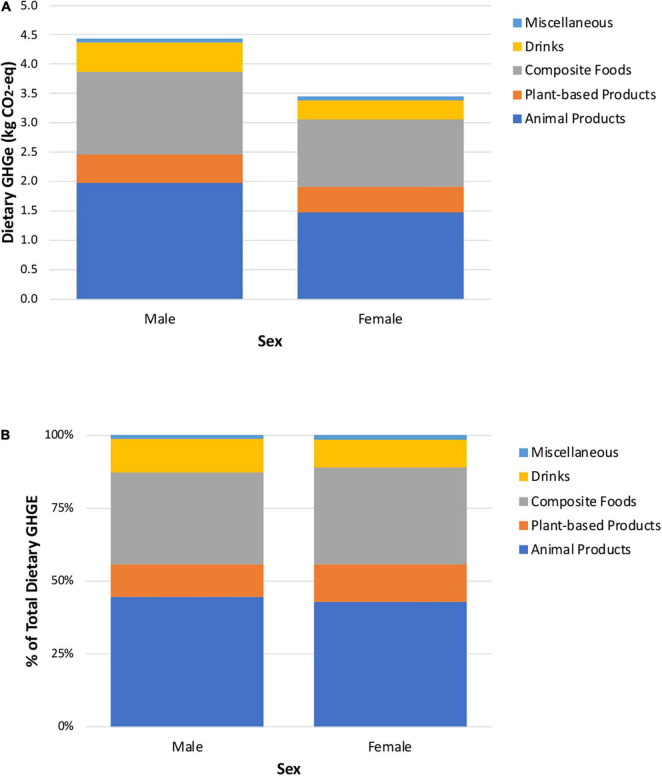
Absolute **(A)** and relative **(B)** contribution of each aggregated food and beverage group to total dietary greenhouse gas emissions, stratified by sex.

**FIGURE 3:**
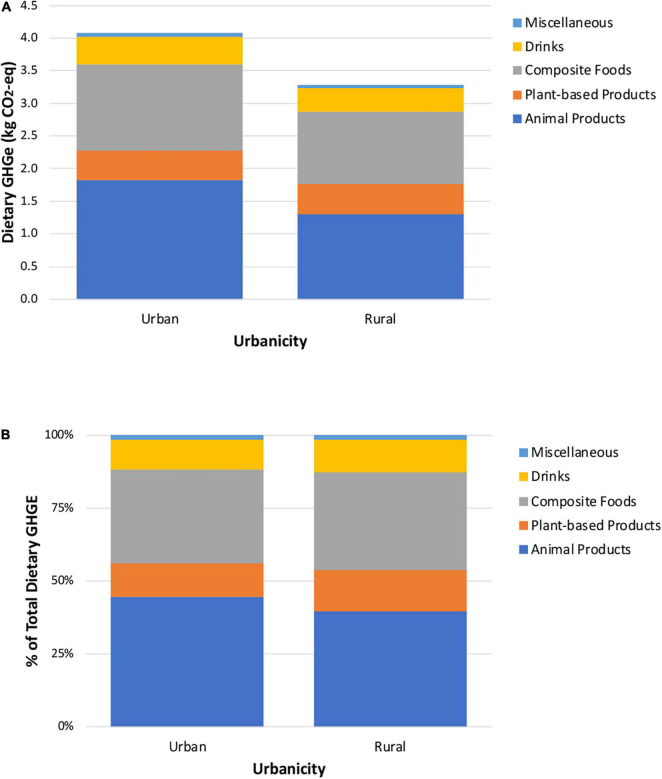
Absolute **(A)** and relative **(B)** contribution of each aggregated food and beverage group to total dietary greenhouse gas emissions, stratified by urbanicity.

Figures 2, 3 present similar graphs of absolute and relative GHGE by sex and urbanicity. Though the diets of men produce higher GHGE than those of women, the proportional contributions are similar for most food groups (Figure 2). The largest differences are that the contribution of beef to total GHGE is greater for men (16.3%) than women (13.5%), as is drinks (11.3 versus 9.5%, respectively). In contrast, dairy, vegetables, and soups, creams and pastas contribute more to total dietary GHGE among women (12.3, 4.5, and 9.6%, respectively) than men (10.4, 3.2, and 7.7%) (Supplementary Figure 2). Results by urbanicity indicate that animal products contribute more to GHGE in urban versus rural areas, in absolute and relative terms. By analyzing the disaggregated food and beverage groups, we found that beef, fast food, and snacks, sweets and desserts contribute more to total dietary GHGE among people in urban areas (15.7, 7.0, and 5.9%, respectively) than those in rural areas (11.5, 2.9, and 4.1%). In contrast, a greater portion of dietary GHGE among people in rural areas is attributable to consumption of corn-based products (16.9% rural versus 11.1% urban) and soups, creams and pastas (9.6% rural versus 8.4% urban).

## Discussion

We found that, overall, diets of Mexican adults and adolescents produce mean total GHGE of 3.9 kg CO_2_-eq/cap/d. This compares to estimates from other studies of 6.8 kg CO_2_-eq/cap/d among Brazilian adults (7), 5.5 kg CO_2_-eq/cap/d for a typical Argentinean diet (6), and 4.7 kg CO_2_-eq/cap/d among adults in the United States (12). One of the main reasons for the lower total dietary GHGE is that the Mexican diet is comprised of lower beef consumption than Brazil and Argentina (19). In Mexico, corn-based products, which are comparatively lower in GHGE, are still a dietary staple. Beef production and consumption play an important role in Argentine culture and each has historically been very high (20). In Brazil, beef consumption nearly doubled from the early 1960s to 2017 (20) and production has increased to the point that Brazil is now the largest beef exporter in the world (21, 22).

Another key finding of this study is that the total carbon footprint of diets varies by demographic and socioeconomic factors. The dietary patterns that contribute to this variation are nuanced and reflect differences in the quantity of calories consumed and differences in the composition of diets. Generally, we found that the diets of more socially disadvantaged groups and regions in the country are more environmentally friendly. The much higher total dietary GHGE of people with higher versus lower educational attainment and non-indigenous versus indigenous language speakers were because those groups consumed a greater number of calories in addition to eating diets with a higher carbon footprint per calorie. In contrast, differences in total dietary GHGE by sex were smaller than might be expected given the much higher energy intake of men relative to women because men’s diets have a lower GHGE load per calorie than diets of women. Adults in different age groups consumed diets with similar carbon footprints per calorie but older adults consumed fewer total calories and thus had lower total GHGE. Energy intake was similar across levels of socioeconomic status and by urbanicity, but those in the higher socioeconomic strata and in urban areas had higher total GHGE because they consumed diets that were less climate friendly on a per-calorie basis, particularly due to a greater contribution of beef and dairy in their diets. These social patterns in diet have important implications for policymakers seeking to improve both health and environmental sustainability. Specifically, different policies, programs, and messaging will likely be required for groups with high dietary GHGE due to the quantity of energy intake versus those who consume relatively high levels of climate unfriendly foods.

Travassos et al. is the only other study in the Latin American region that examined differences in GHGE by social groups (7). They found that total dietary GHGE from 2008 to 2009 in Brazilians aged 10 years and over was greater among men versus women, adults 18–59 versus ≥60 years old, those with lower versus higher levels of education, and those living in rural versus urban areas. We also found that total dietary GHGE was greater among men and adults under 60, but our findings with respect to educational attainment and urbanicity were reversed. Travassos et al. estimated total dietary GHGE of 6.8 kg CO_2_-eq/cap/d in the lowest educational stratum and 6.3 kg CO_2_-eq/cap/d in the highest. In contrast, we estimated dietary GHGE of 3.2 kg CO_2_-eq/cap/d per day in the lowest educational strata and 4.9 kg CO_2_-eq/cap/d in the highest.

Reversed patterns of dietary GHGE between Mexico and Brazil likely relate to social patterns in beef consumption. In Brazil, beef was responsible for 68% of total dietary GHGE and was lowest among those with the highest levels of education. In contrast, we found that beef contributed a much smaller proportion (15%) to the total carbon footprint of the Mexican diet and that beef consumption was greater among those in the higher educational and socioeconomic strata. Differences in the affordability of beef – which is influenced by both beef prices and the income distribution in each country – likely contribute to these social gradients. Mexico’s beef production is one-fifth of Brazil’s, but imports from the United States and Canada – facilitated by a free trade agreement implemented in 1994 – have kept prices relatively low. However, the lower levels of income in Mexico mean that beef has remained unaffordable for a large proportion of the population, especially the 21.4% of the population that is food insecure (23).

Though few studies have examined dietary GHGE in other Latin American countries, dietary data suggest that Brazil and Argentina are likely unique in both their high overall consumption of beef and the inverse relationship between beef consumption and socioeconomic status. For example, a multi-country study of diet in Latin America showed the highest red meat consumption among those in lower socioeconomic strata in Argentina (similar to that observed by Travassos et al. in Brazil), but among those in the highest socioeconomic strata in Chile (similar to what we observed in Mexico) (24). Most other Latin American countries included in the study followed the latter pattern, though differences by socioeconomic status were non-significant. These between-country differences in red meat consumption – which have an outsized impact on total dietary GHGE – are likely driven by access to and affordability of red meat.

The estimation of GHGE has become an important criterion for assessing the environmental sustainability of diets. However, sustainable diets should also be nutritionally adequate (25). The EAT-Lancet Commission proposed a healthy reference diet that considers both human and environmental health (26). Previous studies have found that Mexican adults consume more animal-based foods (particularly red meat, poultry, eggs, and dairy), grains, and added sugars than recommended by the EAT-Lancet diet (19, 27, 28). Our findings support this conclusion. Although beef contributes more to Mexicans’ dietary GHGE than any other food or beverage group, corn-based products, dairy, and drinks also make a non-negligible contribution. As our results show, the diets of the socially disadvantaged groups are more environmentally friendly not because of greater consumption of vegetables or legumes – which would be desirable both from a nutritional and environmental perspective – but rather because of lower consumption of beef and higher consumption of corn products and tortillas. This pattern may further exacerbate the observed shift of obesity in Mexico and other Latin American countries from the rich to the poor (29–32). Therefore, there is room to make the Latin American diet more sustainable, climate-friendly and healthy. Gaining a better understanding of the environmental sustainability and healthfulness of diets will require further analyses that characterize both the carbon footprint of diets (as we did) as well as the nutritional profile of foods.

The study has limitations that should be considered, particularly related to estimation of GHGE. The main data source that we used to estimate GHGE of specific food items was a database intended to calculate the carbon footprint of diets in European countries (15). The food system is largely global, but there are certainly differences in production practices and transportation patterns between Europe and Mexico. That said, transport is responsible for a relatively small portion (∼6%) of the carbon footprint of food, and within-country differences in production practices that affect GHGE are probably as big or bigger than between-country differences. Similarly, we are unable to account for differences between individuals or social groups in methods of food preparation that may lead to substantive differences in GHGE (e.g., LP gas is the dominant cooking fuel in urban areas but fuelwood is dominant in most rural and some peri-urban areas) (33). Importantly, consumption levels captured by food frequency questionnaires may underestimate actual consumption or consumption as measured via other types of instruments (e.g., 24-h dietary recalls). If the ENSANUT data underestimate consumption levels, the GHGE values we calculated will be similarly underestimated (34). Thus, the GHGE levels that we estimate almost certainly have some (unknown) degree of error at the individual level. However, this study provides the first approach using available data to estimate dietary GHGE, overall and by social group in Mexico, a large, highly populated country in the global south. Since dietary GHGE are a major source of global emissions, an important component of future global environmental sustainability strategy should be to develop and routinely update life cycle databases – including for food products – using data from countries in the global south. Our results and interpretations should be taken with caution until GHGE values that consider local food practices are generated.

## Conclusion

The mean total GHGE of the Mexican diet is lower than dietary GHGE estimates from other Latin American countries (i.e., Brazil and Argentina) and the United States. However, similar to these countries, beef contributes more to Mexican dietary GHGE than any other food or beverage group. Other groups, including corn-based products, dairy and drinks, also make important contributions to the carbon footprint of diets in Mexico. Though most studies of the carbon footprint of diets have been based in countries in the global north, meeting environmental sustainability goals will likely require a global strategy that includes assessing the carbon footprints of diet in countries in the global south and using policy levers to maintain or improve the environmental sustainability of diets as these countries grow and develop. Our findings highlight the need to reinforce dietary recommendations that are healthy and sustainable, as well as to consider potential differences across countries and social groups.

## Data Availability Statement

The original contributions presented in the study are included in the article/Supplementary Material, further inquiries can be directed to the corresponding author.

## Ethics Statement

The studies involving human participants were reviewed and approved by the Ethics Committee of the Mexican National Institute of Public Health. Written informed consent to participate in this study was provided by the participants’ legal guardian/next of kin.

## Author Contributions

NL-O and BL were responsible for study design, conception, data preparation, data analysis, and wrote the first draft. NL-O, DS, MB, CP-F, and BL were responsible for interpreting the results. DS, MB, and CP-F revised the draft and made edits. All authors approved the final version.

## Conflict of Interest

The authors declare that the research was conducted in the absence of any commercial or financial relationships that could be construed as a potential conflict of interest.

## Publisher’s Note

All claims expressed in this article are solely those of the authors and do not necessarily represent those of their affiliated organizations, or those of the publisher, the editors and the reviewers. Any product that may be evaluated in this article, or claim that may be made by its manufacturer, is not guaranteed or endorsed by the publisher.

## Footnotes

1 Life cycle analysis refers to the assessment of environmental impacts of a product throughout its life cycle from its raw materials to disposal or recycling.

## Abbreviations

ENSANUT: National Health and Nutrition Survey (for its Spanish acronym)
GHGE: greenhouse gas emissions
LCA: life cycle analyses
SHARP-ID: SHARP Indicators Database.

## References

[1] Crippa M Solazzo E Guizzardi D Monforti-Ferrario F Tubiello FN Leip A. Food systems are responsible for a third of global anthropogenic GHG emissions. *Nat Food.* (2021) 2:198–209. 10.1038/s43016-021-00225-9 37117443

[2] Clune S Crossin E Verghese K. Systematic review of greenhouse gas emissions for different fresh food categories. *J Clean Prod.* (2017) 140:766–83. 10.1016/j.jclepro.2016.04.082

[3] Swinburn BA Kraak VI Allender S Atkins VJ Baker PI Bogard JR The global syndemic of obesity, undernutrition, and climate change: the lancet commission report. *Lancet (Lond Engl).* (2019) 393:791–846. 10.1016/S0140-6736(18)32822-8 30700377

[4] Heller MC Keoleian GA Willett WC. Toward a life cycle-based, diet-level framework for food environmental impact and nutritional quality assessment: a critical review. *Environ Sci Technol.* (2013) 47:12632–47. 10.1021/es4025113 24152032

[5] Hickel J. Quantifying national responsibility for climate breakdown: an equality-based attribution approach for carbon dioxide emissions in excess of the planetary boundary. *Lancet Planet Heal.* (2020) 4:e399–404. 10.1016/S2542-5196(20)30196-0 32918885

[6] Arrieta EM Fischer CG Aguiar S Geri M Fernández RJ Coquet JB The health, environmental, and economic dimensions of future dietary transitions in Argentina. *Sustain Sci.* (2022):1–17. 10.1007/s11625-021-01087-7 [Epub ahead of print]. 35069916 PMC8760564

[7] Travassos GF Antônio da Cunha D Coelho AB. The environmental impact of Brazilian adults’ diet. *J Clean Prod.* (2020) 272:122622. 10.1016/j.jclepro.2020.122622

[8] Aguiar DRD da Costa GN Simões GTC Figueiredo AM. Diet-related greenhouse gas emissions in Brazilian State capital cities. *Environ Sci Policy.* (2021) 124:542–52. 10.1016/j.envsci.2021.07.028

[9] López-Olmedo N Popkin BM Taillie LS. Association between socioeconomic status and diet quality in Mexican men and women: a cross-sectional study. *PLoS One.* (2019) 14:e0224385. 10.1371/journal.pone.0224385 31644595 PMC6808430

[10] Gaona-Pineda EB Martinez-Tapia B Arango-Angarita A Valenzuela-Bravo D Gómez-Acosta LM Shamah-Levy T Food groups consumption and sociodemographic characteristics in Mexican population. *Salud Publica Mex.* (2018) 60:272–82. 10.21149/8803 29746744

[11] Shamah-Levy T Vielma-Orozco E Heredia-Hernández O Romero-Martínez M Mojica-Cuevas J Cuevas-Nasu L *Encuesta Nacional de Salud y Nutricioìn 2018-19.* Cuernavaca: Resultados Nacionales (2020).

[12] Mertens E Kaptijn G Kuijsten A van Zanten H Geleijnse JM van ’t Veer P. SHARP-indicators database towards a public database for environmental sustainability. *Data Br.* (2019) 27:104617. 10.1016/j.dib.2019.104617 31656843 PMC6806457

[13] Romero-Martínez M Shamah-Levy T Vielma-Orozco E Heredia-Hernández O Mojica-Cuevas J Cuevas-Nasu L Encuesta nacional de salud y nutrición 2018-19: metodología y perspectivas. *Salud Publica Mex.* (2019) 61:917–23. 10.21149/11095 31869555

[14] Denova-Gutiérrez E Ramírez-Silva I Rodríguez-Ramírez S Jiménez-Aguilar A Shamah-Levy T Rivera-Dommarco JA. Validity of a food frequency questionnaire to assess food intake in Mexican adolescent and adult population. *Salud Publica Mex.* (2016) 58:617–28. 10.21149/spm.v58i6.7862 28225938

[15] Mertens E Kuijsten A van Zanten HHE Kaptijn G Dofková M Mistura L Dietary choices and environmental impact in four European countries. *J Clean Prod.* (2019) 237:117827. 10.1016/j.dib.2019.104617 31656843 PMC6806457

[16] Heller MC Willits-Smith A Meyer R Keoleian GA Rose D. Greenhouse gas emissions and energy use associated with production of individual self-selected US diets. *Environ Res Lett.* (2018) 13:44004. 10.1088/1748-9326/aab0ac 29853988 PMC5964346

[17] Guzmán-Soria D Taboada-González P Aguilar-Virgen Q Baltierra-Trejo E Marquez-Benavides L. Environmental impact of corn tortilla production: a case study. *Appl Sci.* (2019) 9:4852. 10.3390/app9224852

[18] Instituto Nacional de Salud Pública. *RED PI-DIETA [Internet].* Cuernavaca: Instituto Nacional de Salud Pública (2021).

[19] Shamah-Levy T Gaona-Pineda EB Mundo-Rosas V Méndez Gómez-Humarán I Rodríguez-Ramírez S. [Association of a healthy and sustainable dietary index and overweight and obesity in Mexican adults]. *Salud Publica Mex.* (2020) 62:745–53. 10.21149/11829 33620971

[20] Un Food and Agricultural Organization, Our World in Data. *Meat Consumption by Country, Type, and Year [Internet].* (2021). Available online at: https://ourworldindata.org/meat-production (accessed July 16, 2021).

[21] Economic Research Service Us Department of Agriculture. *Brazil Projected To Outpace Other Top Beef-Exporting Countries Over The Next Decade [Internet].* (2019). Available online at: https://www.ers.usda.gov/data-products/chart-gallery/gallery/chart-detail/?chartId=93444 (accessed June 12, 2021).

[22] da Silva Neto WA Piedade Bacchi MR. Growth of Brazilian beef production: effect of shocks of supply and demand. *Rev Econ Sociol Rural* (2014) 52:209–28. 10.1590/s0103-20032014000200001

[23] Consejo Nacional de Evaluación de la Política de Desarrollo Social. *Evolución de la Población En Pobreza En Materia De Ingresos [Internet].* (2021). Available online at: https://www.coneval.org.mx/Medicion/PublishingImages/Evolucion_carencias_sociales_1990_2015/Pobreza_por_ingresos_1992_2018.PNG (accessed June 23, 2021)

[24] Kovalskys I Rigotti A Koletzko B Fisberg M Gómez G Herrera-Cuenca M Latin American consumption of major food groups: results from the ELANS study. *PLoS One.* (2019) 14:e0225101. 10.1371/journal.pone.0225101 31877144 PMC6932811

[25] Food And Agriculture Organization of the United Nations, World Health Organization. *Sustainable Healthy Diets – Guiding Principles.* Rome: Food And Agriculture Organization of the United Nations (2019).

[26] Willett W Rockström J Loken B Springmann M Lang T Vermeulen S Food in the anthropocene: the EAT–Lancet commission on healthy diets from sustainable food systems. *Lancet.* (2019) 393:447–92. 10.1016/S0140-6736(18)31788-4 30660336

[27] Campirano F López-Olmedo N Salmeron-Castro J. Adherence to the EAT-lancet recommendations in a sample of Mexican health workers. *Curr Dev Nutr.* (2020) 4(Suppl. 2):1383. 10.1093/cdn/nzaa061_011

[28] Castellanos-Gutiérrez A Sánchez-Pimienta TG Batis C Willett W Rivera JA. Toward a healthy and sustainable diet in Mexico: where are we and how can we move forward? *Am J Clin Nutr.* (2021) 113:1177–84. 10.1093/ajcn/nqaa411 33675350

[29] Mendoza A Pérez AE Aggarwal A Drewnowski A. Energy density of foods and diets in Mexico and their monetary cost by socioeconomic strata: analyses of ENSANUT data 2012. *J Epidemiol Community Health.* (2017) 71:713–21. 10.1136/jech-2016-207781 28385691

[30] Batis C Mazariegos M Martorell R Gil A Rivera JA. Malnutrition in all its forms by wealth, education and ethnicity in Latin America: who are more affected? *Public Health Nutr.* (2020) 23 (Suppl. 1):s1–12. 10.1017/S136898001900466X 32900396 PMC10200386

[31] Mazariegos M Auchincloss AH Braverman-Bronstein A Kroker-Lobos MF Ramírez-Zea M Hessel P Educational inequalities in obesity: a multilevel analysis of survey data from cities in Latin America. *Public Health Nutr.* (2021):1–9. 10.1017/S1368980021002457 [Epub ahead of print]. 34167613 PMC7613035

[32] Jiwani SS Carrillo-Larco RM Hernández-Vásquez A Barrientos-Gutiérrez T Basto-Abreu A Gutierrez L The shift of obesity burden by socioeconomic status between 1998 and 2017 in Latin America and the Caribbean: a cross-sectional series study. *Lancet Glob Heal.* (2019) 7:e1644–54. 10.1016/S2214-109X(19)30421-8 31708145 PMC7613084

[33] Berrueta V Serrnano-Medrano M García-Bustamente C Astier M Masera O. Promoting sustainable local development of rural communities and mitigating climate change: the case of Mexico’s Patsari improved cookstove project. *Clim Chang.* (2017) 140:63–77. 10.1007/s10584-015-1523-y

[34] Satija A Yu E Willett WC Hu FB. Understanding nutritional epidemiology and its role in policy. *Adv Nutr.* (2015) 6:5–18. 25593140 10.3945/an.114.007492 PMC4288279

