# Supplementary material for: Greenhouse Gas Emissions Associated With the Mexican Diet: Identifying Social Groups With the Largest Carbon Footprint
Source: Front Nutr. 2022 Mar 31;9:791767. doi: 10.3389/fnut.2022.791767 (PMC9010525; doi:10.3389/fnut.2022.791767)
Supplement: Supplementary file 1 [file Data_Sheet_1.PDF]

Encuesta Nacional de Salud y Nutrición ENSANUT 2019

INEGI

Instituto Nacional de Salud Pública

SALUD
SECRETARÍA DE SALUD

Frecuencia de consumo de alimentos de adolescentes y adultos (12 años o más)

1. DATOS DE IDENTIFICACIÓN

ENTIDAD ______

MUNICIPIO ______

LOCALIDAD ______

AGEB........ -

2. CONTROL DE LA VIVIENDA

UPM ....

NÚMERO DE VIVIENDA SELECCIONADA ....

3. CONTROL DE HOGARES

HOGAR DE EN LA VIVIENDA

4. DATOS DE LA PERSONA SELECCIONADA

NOMBRE ______ N.R

5. RESULTADO FINAL

DÍA MES

RESULTADO ....

CON INFORMACIÓN:

a. Completa
b. Incompleta

6. RESPONSABLES

ESPECIALISTA EN NUTRICIÓN ______ CLAVE

SUPERVISOR(A) ESPECIALISTA ______ CLAVE

7. CONTROL DE PAQUETE

FOLIO DE PAQUETE ______

CONSECUTIVO DEL CUESTIONARIO EN EL PAQUETE ______

CONFIDENCIALIDAD

Conforme a las disposiciones del **Artículo 37, párrafo primero de la Ley del Sistema Nacional de Información Estadística y Geográfica** en vigor: "Los datos que proporcionen para fines estadísticos los Informantes del Sistema a las Unidades en términos de la presente Ley, serán estrictamente confidenciales y bajo ninguna circunstancia podrán utilizarse para otro fin que no sea el estadístico."

OBLIGATORIEDAD

De acuerdo con el **Artículo 45, párrafo primero, de la Ley del Sistema Nacional de Información Estadística y Geográfica** en vigor: "Los informantes del Sistema estarán obligados a proporcionar, con veracidad y oportunidad, los datos e informes que les soliciten las autoridades competentes para fines estadísticos, censales y geográficos, y prestarán apoyo a las mismas".

RESPETO A LAS PERSONAS

De acuerdo al Artículo 13, párrafo primero del Reglamento de la Ley General de Salud en materia de Investigación para la Salud, en vigor; "En toda investigación en la que el ser humano sea sujeto de estudio, deberán prevalecer el criterio del respeto a su dignidad y la protección a sus derechos y bienestar".

ENTREVISTADOR: TRANSCRIBE EL NOMBRE, NÚMERO DE RENGLÓN, SEXO Y EDAD DEL (DE LA) INFORMANTE.

Nombre ______________________ N.R. | | | Sexo H M Edad | | | |

¿En los últimos siete días...			FRECUENCIA DE CONSUMO		PORCIÓN CONSUMIDA	
LEE Y ANOTA NÚMERO EN DÍAS DE LA SEMANA. SI LA RESPUESTA ES 0, PASA A SIGUIENTE ALIMENTO<br>ANOTA TAMAÑO Y NÚMERO DE PORCIÓN DONDE CORRESPONDA			DÍAS DE LA SEMANA	VECES AL DÍA		
			**a) cuántos días comió** (tomó) **usted?**	**b) cuántas veces al día comió** (tomó) **usted?**	**c) Tamaño de porción (*)**	**d) Número de porciones**
	ALIMENTO	PORCIÓN ESTÁNDAR	(0 a 7 días)	(1 hasta 6 veces)		
**1. PRODUCTOS LÁCTEOS**						
1.1	**Leche Liconsa**	1 vaso (240 ml)			E	.
1.2	**Otra leche** (especificar tipo y código: entera, descremada, deslactosada, en polvo, etcétera)					
	a) ____________________	1 vaso (240 ml)			E	.
	b) ____________________	1 vaso (240 ml)			E	.
	c) ____________________	1 vaso (240 ml)			E	.
1.3	**Leche preparada de sabor** (chocolate u otro sabor)	1 vaso (240 ml)			E	.
1.4	**Agregado a la leche:**					
	**a) Azúcar**	1 cucharada cafetera copeteada (10 g)			E	.
	**b) Chocolate u otro saborizante**	1 cucharada cafetera copeteada (10 g)			E	.
1.5	**Queso fresco tipo panela o cottage**	1 rebanada o 2 cucharadas soperas (30 g)			E	.
1.6	**Quesos tipo chihuahua, manchego, gouda, etcétera**	1 rebanada (30 g)			E	.
1.7	**Yogur de vaso:**					
	**a) Entero natural**	1 vaso típico de yogur (150 g)			E	.
	**b) Entero con frutas**	1 vaso típico de yogur (150 g)			E	.
	**c) Bajo en grasa o *light* natural o con fruta** (vitalínea, alpura *light*, lala *light*, etcétera)	1 vaso típico de yogur (150 g)			E	.
1.8	**Yogur para beber:**					
	**a) Entero natural**	1 envase típico (230 g)			E	.
	**b) Entero con fruta**	1 envase típico (230 g)			E	.
	**c) Bajo en grasa o *light* natural o con fruta** (vitalínea, activia 0%, *Siluette*, etcétera)	1 envase típico (230 g)			E	.
1.9	**Danonino o similar**	1 envase (45 g)			E	.
1.10	**Yakult o similares**	1 envase (80 ml)			E	.
**2. FRUTAS**						
2.1	**Plátano**	1 pieza mediana (176 g)				.
2.2	**Plátano frito**	1/2 pieza mediana (113 g)				.
2.3	**Jícama**	1/2 pieza mediana (163 g)				.
2.4	**Naranja o mandarina**	1 pieza grande (206 g)				.
2.5	**Manzana o pera**	1 pieza mediana (140 g)				.

(Continúa)

¿En los últimos siete días... LEE Y ANOTA NÚMERO EN DÍAS DE LA SEMANA. SI LA RESPUESTA ES 0, PASA A SIGUIENTE ALIMENTO ANOTA TAMAÑO Y NÚMERO DE PORCIÓN DONDE CORRESPONDA			FRECUENCIA DE CONSUMO			
			DÍAS DE LA SEMANA	VECES AL DÍA	PORCIÓN CONSUMIDA	
			**a) cuántos días comió** (tomó) **usted?**	**b) cuántas veces al día comió** (tomó) **usted?**	**c) Tamaño de porción (*)**	**d) Número de porciones**
	ALIMENTO	PORCIÓN ESTÁNDAR	(0 a 7 días)	(1 hasta 6 veces)		
2.6	**Melón o sandía**	1 rebanada o 3/4 de taza (115 g)				
2.7	**Guayaba**	1 pieza mediana (75 g)				
2.8	**Mango**	1 pieza mediana (185 g)				
2.9	**Papaya**	1 rebanada mediana (100 g) o 1/2 taza				
2.10	**Piña**	1 rebanada mediana (150 g)				
2.11	**Toronja**	1 pieza chica (270 g)				
2.12	**Fresa**	1 taza o 9 piezas medianas (140 g)			E	
2.13	**Uvas**	10 piezas (60 g)			E	
2.14	**Durazno / melocotón**	1 pieza mediana (50 g)				
2.15	**Frutas en almíbar**	1/2 taza (80 g)			E	
2.16	**Frutas cristalizadas o secas**	1/4 taza (25 g)			E	

**3. VERDURAS**						
3.1	**Tortitas de verduras capeadas**	1 pieza (72 g)			E	
3.2	**Jitomate**	1/2 pieza chica (30 g) en ensalada				
3.3	**Hojas verdes** (acelgas, espinacas, quelites)	1/2 plato (85 g) cocidas o 1 plato crudas			E	
3.4	**Chayote**	1/4 pieza chica (50 g) o 1/3 taza				
3.5	**Zanahoria**	1 pieza chica o 1/2 taza (50 g)				
3.6	**Calabacita**	1/2 pieza mediana (50 g)				
3.7	**Brócoli o coliflor**	1/4 taza (35 g)			E	
3.8	**Col**	1/4 taza (35 g)			E	
3.9	**Ejotes**	1/4 taza o 5 piezas (30 g)			E	
3.10	**Elote**	1/2 pieza chica (50 g)				
3.11	**Lechuga**	1/2 taza o 1 hoja (30 g)			E	
3.12	**Nopales**	1 pieza grande (100 g)				
3.13	**Pepino**	1/2 pieza grande (150 g)				
3.14	**Aguacate**	1 rebanada o 1 pieza de criollo chico (33 g)				
3.15	**Chile poblano**	Una pieza mediana o 1/3 taza (80 g)				
3.16	**Cebolla, por ejemplo, en ensaladas, antojitos o comida rápida**	1 cucharada sopera o 3 rodajas (7 g)				

(Continúa)

¿En los últimos siete días... LEE Y ANOTA NÚMERO EN DÍAS DE LA SEMANA. SI LA RESPUESTA ES 0, PASA A SIGUIENTE ALIMENTO ANOTA TAMAÑO Y NÚMERO DE PORCIÓN DONDE CORRESPONDA			FRECUENCIA DE CONSUMO			
			DÍAS DE LA SEMANA	VECES AL DÍA	PORCIÓN CONSUMIDA	
			**a) cuántos días comió** (tomó) **usted?**	**b) cuántas veces al día comió** (tomó) **usted?**	**c) Tamaño de porción (*)**	**d) Número de porciones**
	ALIMENTO	PORCIÓN ESTÁNDAR	(0 a 7 días)	(1 hasta 6 veces)		
3.17	**Verduras envasadas como chícharo, zanahoria, champiñones y ejotes**	1/3 taza o 1 lata pequeña			E	.
3.18	**Verduras congeladas como chícharo, zanahoria, brócoli, coliflor, ejotes**	1/3 taza			E	.
**4. COMIDA RÁPIDA**						
4.1	**Torta o sándwich**	1 pieza mediana (130 g)				.
4.2	**Hamburguesa**	1 pieza Mediana (240 g)				.
4.3	**Pizza**	1 rebanada chica (92 g)				.
4.4	**Hot dog**	1 pieza mediana (110 g)				.
**5. CARNES, EMBUTIDOS Y HUEVO**						
5.1	**Carne de puerco**	1 bistec mediano (90 g)				.
5.2	**Carne de res**	1 bistec mediano (90 g)				.
5.3	**Carne de res seca** (machaca)	1 plato (80 g)			E	.
5.4	**Longaniza o chorizo**	1/2 trozo (30 g)			E	.
5.5	**Salchicha o jamón de puerco, pavo o combinado, y/o mortadela** (aparte de en torta, sándwich o hot dog)	1 pieza de salchicha o 1 reb. de jamón (30 g)			E	.
5.6	**Pollo** **a) Pierna, muslo o pechuga**	1 pieza (pierna, muslo) o 1/2 pieza de pechuga chica (90 g)				.
	**b) Ala, patas**	1 pieza de ala, 2 piezas de patas (70 g)				.
	**c) Higadito o molleja**	1 pieza de higadito o molleja (30 g)				.
5.7	**Huevo** **a) Tibio o cocido**	1 pieza entera de huevo tibio o cocido (62 g)			E	.
	**b) Frito, estrellado o revuelto**	1 pieza entera de huevo frito, estrellado o revuelto (55 g)			E	.
**6. PESCADOS Y MARISCOS**						
6.1	**Pescado fresco**	1 filete mediano o mojarra chica (90 g)				.
6.2	**Pescado seco** (charalitos, bacalao)	1 plato (80 g)			E	.
6.3	**Atún y sardina** (en tomate, agua o aceite)	1/4 lata o (40 g)			E	.
6.4	**Algún marisco** (camarón, ostiones, etcétera)	1 plato (100 g)			E	.

(Continúa)

¿En los últimos siete días...<br><br>LEE Y ANOTA NÚMERO EN DÍAS DE LA SEMANA. SI LA RESPUESTA ES 0, PASA A SIGUIENTE ALIMENTO<br>ANOTA TAMAÑO Y NÚMERO DE PORCIÓN DONDE CORRESPONDA			FRECUENCIA DE CONSUMO			
			DÍAS DE LA SEMANA	VECES AL DÍA	PORCIÓN CONSUMIDA	
	ALIMENTO	PORCIÓN ESTÁNDAR	**a) cuántos días comió** (tomó) **usted?**<br>(0 a 7 días)	**b) cuántas veces al día comió** (tomó) **usted?**<br>(1 hasta 6 veces)	**c) Tamaño de porción (*)**	**d) Número de porciones**
**7. LEGUMINOSAS**						
7.1	**Frijoles preparados en casa:**					
	**a) De la olla**	1/2 plato o 1/2 taza (50 g)			E	.
	**b) Refritos**	1/2 plato o 1/2 taza (50 g)			E	.
7.2	**Frijoles envasados o de lata:**					
	**a) De la olla**	1/2 plato o 1/2 taza (50 g)			E	.
	**b) Refritos**	1/2 plato o 1/2 taza (50 g)			E	.
7.3	**Lenteja, garbanzo, haba amarilla o alubia**	1 plato o 1 taza (100 g)			E	.

**8. CEREALES Y TUBÉRCULOS**						
8.1	**Arroz guisado**	1 taza o 1 plato (100 g)			E	.
8.2	**Pan blanco**	2 rebanadas o 1 bolillo (70 g)			E	.
8.3	**Pan integral**	2 rebanadas o 1 bolillo (70 g)			E	.
8.4	**Pan dulce** (excepto donas y churros)	1 pieza (70 g)			E	.
8.5	**Donas y churros de panadería**	1 pieza (70 g)			E	.
8.6	**Galletas saladas**	4 piezas (20 g)			E	.
8.7	**Papas**<br>**a) Cocida**	1/2 pieza mediana cocida (40 g)			E	.
	**b) Frita o tortita de papa**	1/2 pieza mediana frita o tortita de papa (40 g)			E	.
8.8	**Cereal de caja:**					
	**a) Chocolate** (Chocozucaritas, chocokrispis)	1 taza (seco 30 g)			E	.
	**b) *Light*/cuidado de la figura** (Special K)	1 taza (seco 30 g)			E	.
	**c) Hojuela endulzada** (Zucaritas)	1 taza (seco 30 g)			E	.
	**d) Básico** (Corn Flakes, arroz inflado sin sabor)	1 taza (seco 30 g)			E	.
	**e) Variedades** (Apple jacks, honey smacks, corn pops)	1 taza (seco 30 g)			E	.
	**f) Sabor a frutas** (Froot loops)	1 taza (seco 30 g)			E	.
	**g) Fibra** (All Bran)	1 taza (seco 30 g)			E	.
	**h) Especialidades** (Crusli)	1 taza (seco 30 g)			E	.
	**i) Multi ingredientes** (Extra)	1 taza (seco 30 g)			E	.

**9. PRODUCTOS DE MAÍZ**						
9.1	**Antojitos sin carne como sopes, quesadillas, tlacoyos, gorditas, y enchiladas (NO TACOS):**					
	**a) Sin freír**	100 g			E	.
	**b) Fritos**	100 g			E	.

(Continúa)

¿En los últimos siete días... LEE Y ANOTA NÚMERO EN DÍAS DE LA SEMANA. SI LA RESPUESTA ES 0, PASA A SIGUIENTE ALIMENTO. ANOTA TAMAÑO Y NÚMERO DE PORCIÓN DONDE CORRESPONDA			FRECUENCIA DE CONSUMO			
			DÍAS DE LA SEMANA	VECES AL DÍA	PORCIÓN CONSUMIDA	
	ALIMENTO	PORCIÓN ESTÁNDAR	**a) cuántos días comió** (tomó) **usted?** (0 a 7 días)	**b) cuántas veces al día comió** (tomó) **usted?** (1 hasta 6 veces)	**c) Tamaño de porción (*)**	**d) Número de porciones**
9.2	**Antojitos con res, cerdo, pollo, vísceras, etc., como tacos, quesadillas, tlacoyos, enchiladas, gorditas:**					
	**a) Sin freír**	100 g			E	.
	**b) Fritos**	100 g			E	.
9.3	**Pozole** (todos tipos)	1 plato (100 g)			E	.
9.4	**Tamal** (todos tipos)	1 pieza (200 g)			E	.
9.5	**Atole de maíz**					
	**a) Atole con agua**	1 taza (240 ml)			E	.
	**b) Atole con leche** (aparte de la reportada en el apartado de "Productos lácteos"). Especificar tipo de leche y código: entera, descremada, deslactosada, en polvo, etcétera.					
	b1) ____________________	1 taza (240 ml)			E	.
	b2) ____________________	1 taza (240 ml)			E	.
	b3) ____________________	1 taza (240 ml)			E	.

**10. BEBIDAS**						
10.1	**Refresco normal**	1 vaso (240 ml)			E	.
10.2	**Refresco dieta**	1 vaso (240 ml)			E	.
10.3	**Café:**					
	**a) Café sin azúcar**	1 taza (240 ml)			E	.
	**b) Azúcar agregada al café**	1 cucharada cafetera copeteada (10 g)			E	.
	**c) Leche agregada al café** (aparte de la reportada en el apartado de "Productos lácteos"). Especificar tipo de leche y código: entera, descremada, deslactosada, en polvo, etcétera.					
	c1) ____________________	1 taza (240 ml)			E	.
	c2) ____________________	1 taza (240 ml)			E	.
	c3) ____________________	1 taza (240 ml)			E	.
	**d) Sustituto de crema agregada al café**	1 cucharada sopera			E	.
10.4	**Té o infusión:**					
	**a) Té sin azúcar**	1 taza (240 ml)			E	.
	**b) Azúcar agregada al té**	1 cucharada cafetera copeteada (10 g)			E	.
10.5	**Jugos naturales sin azúcar**	1 vaso (240 ml)			E	.
10.6	**Jugos naturales con azúcar**	1 vaso (240 ml)			E	.
10.7	**Aguas de fruta natural con azúcar**	1 vaso (240 ml)			E	.
10.8	**Aguas de fruta natural sin azúcar**	1 vaso (240 ml)			E	.

(Continúa)

¿En los últimos siete días...<br>LEE Y ANOTA NÚMERO EN DÍAS DE LA SEMANA. SI LA RESPUESTA ES 0, PASA A SIGUIENTE ALIMENTO<br>ANOTA TAMAÑO Y NÚMERO DE PORCIÓN DONDE CORRESPONDA			FRECUENCIA DE CONSUMO			
			DÍAS DE LA SEMANA	VECES AL DÍA	PORCIÓN CONSUMIDA	
	ALIMENTO	PORCIÓN ESTÁNDAR	**a) cuántos días comió** (tomó) **usted?**<br>(0 a 7 días)	**b) cuántas veces al día comió** (tomó) **usted?**<br>(1 hasta 6 veces)	**c) Tamaño de porción (*)**	**d) Número de porciones**
10.9	**Bebidas o aguas de sabor industrializadas sin azúcar** (incluyendo dietéticas como Clight, Be-light, etcétera)	1 vaso (240 ml)			E	
10.10	**Bebidas o aguas de sabor industrializadas con azúcar** (frutsi, bonafina)	1 vaso (240 ml)			E	
10.11	**Néctares de frutas o pulpa de frutas industrializados con azúcar** (boing, jumex)	1 vaso (240 ml)			E	
10.12	**Agua sola**	1 vaso (240 ml)			E	
10.13	**Bebidas alcohólicas**	1 vaso (240 ml) de cerveza, vino, pulque, cuba o copa solo con tequila, mezcal u otro			E	

**11. BOTANAS, DULCES Y POSTRES**						
11.1	**Chocolate**	1 trozo o 1 cucharada sopera (10 g)			E	
11.2	**Dulce** (caramelos, paletas)	1 pieza (30 g)			E	
11.3	**Dulces enchilados** (miguelitos, tamarindos)	1 pieza (30 g)			E	
11.4	**Frituras** (todos tipos, incluyendo cacahuates japoneses)	1 paquete individual o bolsa chica (35 g)			E	
11.5	**Paletas y dulces de malvavisco** (paleta payaso, bubu-lu-bu)	2 piezas pequeñas o 1 pieza grande (40 g)			E	
11.6	**Gelatina, flan**	1 pieza o rebanada (125 g)			E	
11.7	**Pastel o pay**	1 rebanada mediana (125 g)			E	
11.8	**Helado, nieves y paletas de agua**	1 pza o 1 bola (80 g)			E	
11.9	**Helado y paletas de leche**	1 pza o 1 bola (80 g)			E	
11.10	**Cacahuates, habas o pepitas**	1 puño (de la mano) (35 g)			E	
11.11	**Palomitas de maíz de microondas o del cine** (todos tipos, excepto acarameladas)	1 bolsa mediana (100 g)			E	
11.12	**Pastelillos y donas industrializadas**	1 pieza (70 g)			E	
11.13	**Galletas dulces** (todos tipos)	2 piezas (32 g)			E	
11.14	**Barras de cereal**	1 pieza (25 g)			E	

**12. SOPAS, CREMAS Y PASTAS**						
12.1	**Caldo de pollo, res o verduras** (solo caldo)	1 taza (240 ml)			E	
12.2	**Sopa o caldo con verduras**	1 plato (240 ml)			E	
12.3	**Sopa de pasta**<br>**a) Sopa caldosa**	1/2 plato o 1/2 taza sopa caldosa (50 g)			E	
	**b) Sopa seca**	1/2 plato sopa seca (50 g)			E	

(Continúa)

¿En los últimos siete días... LEE Y ANOTA NÚMERO EN DÍAS DE LA SEMANA. SI LA RESPUESTA ES 0, PASA A SIGUIENTE ALIMENTO ANOTA TAMAÑO Y NÚMERO DE PORCIÓN DONDE CORRESPONDA			FRECUENCIA DE CONSUMO			
			DÍAS DE LA SEMANA	VECES AL DÍA	PORCIÓN CONSUMIDA	
	ALIMENTO	PORCIÓN ESTÁNDAR	**a) cuántos días comió** (tomó) **usted?** (0 a 7 días)	**b) cuántas veces al día comió** (tomó) **usted?** (1 hasta 6 veces)	**c) Tamaño de porción (*)**	**d) Número de porciones**
12.4	**Crema de verduras**	1 plato (240 ml)	__	__	E	__ . __ __
12.5	**Sopas instantáneas**	1 vaso (64 g)	__	__	E	__ . __ __

**13. MISCELÁNEOS**						
13.1	**Limón, por ejemplo, en ensaladas, caldos, o carnes**		__	__		1
13.2	**Cebolla, por ejemplo, en salsas, o caldillos** (molida o entera)		__	__		1
13.3	**Chiles frescos, por ejemplo, en salsas, tacos, guisados** (molido o entero)		__	__		1
13.4	**Chiles envasados o enlatados, por ejemplo, en el sándwich, torta, guisados.**		__	__		1
13.5	**Chile seco, por ejemplo, en salsas, tacos, guisados** (molido o entero)		__	__		1
13.6	**Tomate verde y jitomate, por ejemplo, en salsas, tacos, o guisados** (molido o entero)		__	__		1
13.7	**Azúcar** (aparte de la agregada a las bebidas, leche, té, café, agua de frutas), **por ejemplo, en fresas o plátanos con crema**	1 cucharada sopera (10 g)	__	__	E	__ . __ __
13.8	**Margarina**	1 cucharada sopera (10 g)	__	__	E	__ . __ __
13.9	**Mantequilla**	1 cucharada sopera (10 g)	__	__	E	__ . __ __
13.10	**Mayonesa**	1 cucharada sopera (10 g)	__	__	E	__ . __ __
13.11	**Crema**	1 cucharada sopera (10 g)	__	__	E	__ . __ __
13.12	**Manteca vegetal**	1 cucharada sopera (10 g)	__	__	E	__ . __ __
13.13	**Manteca animal** (cerdo o pollo)	1 cucharada sopera (10 g)	__	__	E	__ . __ __
13.14	**Sal o condimento con sal agregada a sus alimentos**	(Sal de mesa, sal con ajo, sal con cebolla)	__	__	Cantidad agregada __ a. Poca b. Moderada c. Mucha	

(Continúa)

¿En los últimos siete días... LEE Y ANOTA NÚMERO EN DÍAS DE LA SEMANA. SI LA RESPUESTA ES 0, PASA A SIGUIENTE ALIMENTO ANOTA TAMAÑO Y NÚMERO DE PORCIÓN DONDE CORRESPONDA			FRECUENCIA DE CONSUMO		PORCIÓN CONSUMIDA	
			DÍAS DE LA SEMANA	VECES AL DÍA		
	ALIMENTO	PORCIÓN ESTÁNDAR	**a) cuántos días comió** (tomó) **usted?** (0 a 7 días)	**b) cuántas veces al día comió** (tomó) **usted?** (1 hasta 6 veces)	**c) Tamaño de porción (*)**	**d) Número de porciones**
13.15	**Salsas y aderezos agregados a sus alimentos:**					
	**a) Cátsup**		|__|	|__|	Cantidad agregada |__| a. Poca b. Moderada c. Mucha	
	**b) Salsa picante para botana agregada a sus alimentos**		|__|	|__|	Cantidad agregada |__| a. Poca b. Moderada c. Mucha	
	**c) Salsa de soya, salsa inglesa o sazonadores líquidos agregados a sus alimentos**		|__|	|__|	Cantidad agregada |__| a. Poca b. Moderada c. Mucha	

(*) TAMAÑO DE PORCIÓN:
CARNES: Muy chico (MC); Chico (C); Mediano (M); Grande (G); Muy Grande (MG); Estándar (E).
FRUTAS, VERDURAS, COMIDA RÁPIDA: Chico (C); Mediano (M); Grande (G); Estándar (E).

14. TORTILLAS

¿En los últimos siete días... LEE Y ANOTA NÚMERO EN DÍAS. SI LA RESPUESTA ES 0, PASA A SIGUIENTE ALIMENTO		FRECUENCIA DE CONSUMO	
14.1	**aparte de las tortillas consumidas en enchiladas u otro antojito, ¿cuántos días comió tortilla de maíz?**	a) Días de consumo (0 a 7 días)	**b) ¿Cuántas tortillas comió cada día que las consumió?**
	**a) de nixtamal** (hecho en casa)	|__|	|__|__| . |__|__|
	**b) de harina MASECA o MINSA** (hecha en casa)	|__|	|__|__| . |__|__|
	**c) de masa** (comprada) **o de tortillería**	|__|	|__|__| . |__|__|
14.2	**¿Cuántos días comió tortillas de harina de trigo?**	|__|	|__|__| . |__|__|
14.3	**PESO PROMEDIO DE TORTILLA DE MAÍZ**	GRAMOS:	|__|__|__| . |__|
14.4	**PESO PROMEDIO DE TORTILLA DE TRIGO**	GRAMOS:	|__|__|__| . |__|

15. CANTIDAD DE CONSUMO REPORTADA

¿Considera usted que el consumo que reportó fue semejante a lo que come normalmente?, o ¿fue mayor o menor? (ya que su consumo pudo haber variado por enfermedad y comer poco o haber tenido fiesta y comer más)

CRUZA UN CÓDIGO

Igual 1
Mayor 2
Menor 3

**¿En los últimos siete días...** LEE Y ANOTA NÚMERO EN DÍAS DE LA SEMANA. SI LA RESPUESTA ES 0, PASA A SIGUIENTE ALIMENTO<br>ANOTA TAMAÑO Y NÚMERO DE PORCIÓN DONDE CORRESPONDA			FRECUENCIA DE CONSUMO		PORCIÓN CONSUMIDA	
			DÍAS DE LA SEMANA	VECES AL DÍA		
	ALIMENTO	PORCIÓN ESTÁNDAR	**a) cuántos días comió** (tomó) **usted?** (0 a 7 días)	**b) cuántas veces al día comió** (tomó) **usted?** (1 hasta 6 veces)	**c) Tamaño de porción (*)**	**d) Número de porciones**
**16. CONSUMO DE SUPLEMENTOS**						
16.1	**Nutrivida del Programa PROSPERA**	1 Tableta	|__|	|__|	|E|	|__|.|__|__|
16.2	**Algún suplemento y/o complemento alimenticio**					
	Codifica tipo de suplemento. Si no está en el catálogo, especifica (marca, nombre, vitaminas, minerales, etcétera)<br>a) |__|__|__| ____________	CRUZA UN CÓDIGO<br>Tabletas, grageas, comprimidos, o cápsulas (1 pieza).......... 1<br>Polvo (1 cucharada).......... 2<br>Líquido (1 cucharada o 10 ml)..... 3<br>Envase (1 envase 237 ml).......... 4<br>Inyectable (1 ampolleta).......... 5<br>Gotas (1/2 gotero o 3 ml).......... 6	|__|	|__|	|E|	|__|.|__|__|
	b) |__|__|__| ____________	CRUZA UN CÓDIGO<br>Tabletas, grageas, comprimidos, o cápsulas (1 pieza).......... 1<br>Polvo (1 cucharada).......... 2<br>Líquido (1 cucharada o 10 ml)..... 3<br>Envase (1 envase 237 ml).......... 4<br>Inyectable (1 ampolleta).......... 5<br>Gotas (1/2 gotero o 3 ml).......... 6	|__|	|__|	|E|	|__|.|__|__|
	c) |__|__|__| ____________	CRUZA UN CÓDIGO<br>Tabletas, grageas, comprimidos, o cápsulas (1 pieza).......... 1<br>Polvo (1 cucharada).......... 2<br>Líquido (1 cucharada o 10 ml)..... 3<br>Envase (1 envase 237 ml).......... 4<br>Inyectable (1 ampolleta).......... 5<br>Gotas (1/2 gotero o 3 ml).......... 6	|__|	|__|	|E|	|__|.|__|__|

OBSERVACIONES:
