# Supplementary material for: Greenhouse Gas Emissions Associated With the Mexican Diet: Identifying Social Groups With the Largest Carbon Footprint
Source: Front Nutr. 2022 Mar 31;9:791767. doi: 10.3389/fnut.2022.791767 (PMC9010525; doi:10.3389/fnut.2022.791767)
Supplement: Supplementary file 3 [file Data_Sheet_3.docx]

A

B

**Figure S1.** Absolute (A) and relative (B) contribution of each disaggregated food and beverage group to total dietary greenhouse gas emissions, stratified by socioeconomic status.

A

B

**Figure S2.** Absolute (A) and relative (B) contribution of each disaggregated food and beverage group to total dietary greenhouse gas emissions, stratified by sex.

A

B

**Figure S3.** Absolute (A) and relative (B) contribution of each disaggregated food and beverage group to total dietary greenhouse gas emissions, stratified by urbanicity.
